# Identifying Strong Neoantigen MHC-I/II Binding Candidates for Targeted Immunotherapy with SINE

**DOI:** 10.3390/ijms26010205

**Published:** 2024-12-29

**Authors:** Joseph Bendik, Andrea Castro, Joseph Califano, Hannah Carter, Theresa Guo

**Affiliations:** 1 Moores Cancer Center, University of California San Diego, San Diego, CA 92037, USA; 2 Division of Medical Genetics, Department of Medicine, University of California San Diego, San Diego, CA 92093, USA; 3 Gleiberman Head and Neck Cancer Center, University of California San Diego, San Diego CA 92037, USA; 4 Division of Otolaryngology-Head and Neck Surgery, Department of Surgery, University of California San Diego, San Diego, CA 92037, USA

**Keywords:** alternative splicing, head and neck cancer, melanoma, neoantigen, targeted immunotherapy response, software

## Abstract

The discovery of tumor-derived neoantigens which elicit an immune response through major histocompatibility complex (MHC-I/II) binding has led to significant advancements in immunotherapy. While many neoantigens have been discovered through the identification of non-synonymous mutations, the rate of these is low in some cancers, including head and neck squamous cell carcinoma. Therefore, the identification of neoantigens through additional means, such as aberrant splicing, is necessary. To achieve this, we developed the splice isoform neoantigen evaluator (SINE) pipeline. Our tool documents peptides present on spliced or inserted genomic regions of interest using Patient Harmonic-mean Best Rank scores, calculating the MHC-I/II binding affinity across the complete human leukocyte antigen landscape. Here, we found 125 potentially immunogenic events and 9 principal binders in a cohort of head and neck cancer patients where the corresponding wild-type peptides display no MHC-I/II affinity. Further, in a melanoma cohort of patients treated with anti-PD1 therapy, the expression of immunogenic splicing events identified by SINE predicted response, potentially indicating the existence of immune editing in these tumors. Overall, we demonstrate SINE’s ability to identify clinically relevant immunogenic neojunctions, thus acting as a useful tool for researchers seeking to understand the neoantigen landscape from aberrant splicing in cancer.

## 1. Introduction

In recent years, the immune checkpoint blockade, especially the development of programed cell death receptor (PD-1) inhibitors, has emerged as a revolution in cancer therapy. Often, tumor cells can express higher levels of programmed cell death receptor–ligand 1 (PD-L1), which then binds to PD-1 on T-Cells, suppressing immune system activity [1]. The development of checkpoint inhibitor drugs such as nivolumab and pembrolizumab have allowed for the blockade of these checkpoints, opening the door for immune-mediated tumor killing [2]. However, many patients remain unresponsive to these treatments. The causes are postulated to be due to a variety of potential factors such as a lack of tumor antigen recognition, defective effector T cell function, and/or microenvironment differences [3,4].

Because of these limitations, the discovery of novel tumor-derived neoantigens can provide insight for both predicting responders and the development of novel targets to enhance treatment efficacy. Neoantigens are proteins that are uniquely produced by tumor cells with the ability to be presented by the immune system through the major histocompatibility complex (MHC-I/II) and elicit an immune response. In the past, neoantigens have been traditionally identified from non-synonymous genomic mutations [5,6]. However, this likely provides a limited view of the true neoantigen landscape as neoantigens have now been reported in other genetic alterations, including gene fusions, indels, intron retention events, and splicing alterations [7,8,9,10,11]. Understanding neoantigens derived from these alternate sources is especially relevant in low-tumor-mutational-burden tumors, such as head and neck squamous cell carcinoma or ovarian cancer [12,13,14].

In cancer, one relatively understudied method for neoantigen detection that demonstrates promise is aberrant splicing [11,14,15,16]. The differential arrangement, inclusion, or exclusion of exons leads to a significant alteration of peptide sequences and an increase in protein diversity that can represent a new source for neoantigens [17]. Comprehensive studies on data from The Cancer Genome Atlas (TCGA) have shown that aberrant novel splicing events with no expression in normal tissue occur frequently across several cancer types [11]. Additionally, in cases such as head and neck cancer, alternative splicing has been demonstrated to produce functionally active variants of *AKT3*, *DOCK5*, and *GSN* [18,19,20]. This is particularly important as head and neck cancer patients often display a low tumor mutational burden but a high splice burden, thus demonstrating the importance of looking not just at somatic mutation derived neoantigens, but splice-derived variants as well [11,12].

To help researchers discover splice-derived neoantigens, a few open-source software tools have been developed, including ASNEO (v.n/a), NeoSplice (v.0.0.3), and SNAF (v.0.7.0) [21,22,23]. ASNEO takes in junctions with a high percent spliced in rate and inserts them into the hg19 reference isoforms to generate novel isoforms that undergo one-frame translation. NeoSplice uses a kmer search with the Burrows–Wheeler transform and CIGAR string parsing methodologies to generate neoantigen predictions from paired tumor and normal samples. SNAF utilizes a combination of deep learning and Bayesian probabilistic modeling to predict immunogenicity and rank the neoantigen’s tumor specificity. However, ASNEO’s required use of the human genome build 19 (hg19) leads to a diminished ability to detect novel events, and the matching tumor and normal sample requirement by NeoSplice can limit the analysis of real-world samples. Additionally, when evaluating MHC-I binding affinity, these programs only consider individual human leukocyte antigen (HLA) alleles and not their combination. Previously, we have shown that the combination of a patient’s MHC-I genotypes directly influences the probability for the tumor to acquire and present a recurrent mutation [24]. For these reasons, we developed SINE (splice isoform neoantigen evaluator) to provide an up-to-date, generalizable neoantigen discovery pipeline that accounts for the overall MHC-I and MHC-II binding probability using the Patient Harmonic-mean Best Rank (PHBR) scoring method. This method uses an aggregation of best rank binding affinity scores across all of a patient’s MHC alleles, allowing for a more accurate representation of the patient’s peptide presentation ability. SINE is also able to identify and calculate the binding affinity of paired wild-type (WT) junction peptides in normal samples to help with neoantigen validation.

Previously, we developed the algorithm OutSplice to detect novel cancer-specific alternative splicing events. Herein, we utilized OutSplice to identify tumor-specific splicing events in a previously published oropharyngeal squamous cell carcinoma (OPSCC) dataset, and apply SINE to identify and characterize putative immunogenic splice-derived neoantigens [25]. These events were compared to outputs from other existing splicing neoantigen detection software (ASNEO and SNAF). In addition, we applied SINE to a cohort of melanoma patients treated with anti-PD1 therapy to show that splice-derived neoantigens identified by SINE undergo immune editing specifically in responders [26]. To help evaluate SINE’s capabilities, we also applied our tool on a subset of skin cutaneous melanoma (SKCM) patient data from The Cancer Genome Atlas (TCGA) and compared the results to splice-derived neoantigens with a previously performed mass-spectrometry (MS) validation [23]. These data demonstrate the ability of SINE to identify clinically significant immunogenic splice-derived neoantigens that can delineate patients responding to anti-PD1 therapy.

## 2. Results

### 2.1. OPSCC Cohort

SINE was run on an OPSCC dataset of 47 tumors using junction events with outlier expression relative to 25 normal controls. In total, after running SINE, we found 125 events to contain peptide sequences with relevant MHC-I binding ability (PHBR < 2) (Table S1). Additionally, we discovered 19 events that contain peptide sequences with a strong (<0.5 PHBR) average binding affinity score. However, from the total list, we found that: 60 of these events did not occur in a majority of the samples, 30 displayed significant outlier underexpression in the tumor, 26 had a WT alternative with stronger MHC-I binding, and 53 events had no WT alternative at all. Therefore, to create a list of the top potential neoantigen candidates, we filtered these 125 aberrant events based on the following criteria: has significant outlier overexpression based on OutSplice analysis, has an alternative WT splicing event with a larger PHBR score, and occurs in >50% of the tumor samples.

From this, we identified 12 overexpressed events in tumor tissue across 11 genes to have MHC-I binding and a binding score lower than the WT on average (Table 1). Furthermore, two events overexpressed in the tumor tissues on the *MKNK2* and *FGFR1OP* genes displayed very strong affinity for MHC-I and a corresponding WT event without MHC-I binding affinity. We also found that in six of the events, while the average PHBR score only indicated weak MHC-I binding (PHBR > 0.5 but <2), the peptides spanning the WT alternative junction still displayed no ability to bind to the patient’s HLA types. All of these events occurred in the majority of the tumor tissue samples, with a median occurrence of 98% for the immunogenic events with no MHC-I binding WT alternative. Using OutSplice’s outlier functions, these events were highly prevalent as outliers, showing significant upregulation in expression compared to normal tissue. The median outlier prevalence was 53.2%, with the lowest prevalence being 31.9%. PHBR scores for the OPSCC cohort were also calculated for MHC-II binding. Here, while 5 strong binders and 53 overall relevant binders were detected, only 1 event on the *CENPW* gene met our above stringent criteria (Table S1). This resulted in nine overall slicing events without a WT binding alternative that met our criteria for principal MHC-I/II binders.

### 2.2. SINE Comparison to ASNEO and SNAF

We performed a parallel analysis of the OPSCC cohort using SNAF and ASNEO. Out of the 73,941 splice events detected by AltAnalyze, the SNAF tool identified 2607 alternative splicing events where at least 1 of the 47 samples displayed peptide immunogenicity based on their NetMHCpan score and DeepImmuno percentile [27]. In contrast, SINE narrowed down a custom list of 344 junctions with outlier expression to a list of 194 immunogenic events where at least 1 sample displayed immunogenicity at the PHBR level. From these events, we found three splicing events on the *STAG3*, *STEAP1B*, and *MRC2* genes that overlap both detection tools’ standards for immunogenicity warranting further wet lab testing (Table 2). The ASNEO tool identified 279 splicing events where at least 1 sample displayed peptide immunogenicity based on their self-described immunogenicity score. When using ASNEO, we were unable to find any consensus neoantigen predictions between it and SINE or SNAF.

### 2.3. Immunotherapy-Treated Melanoma Dataset

**Figure 1:**
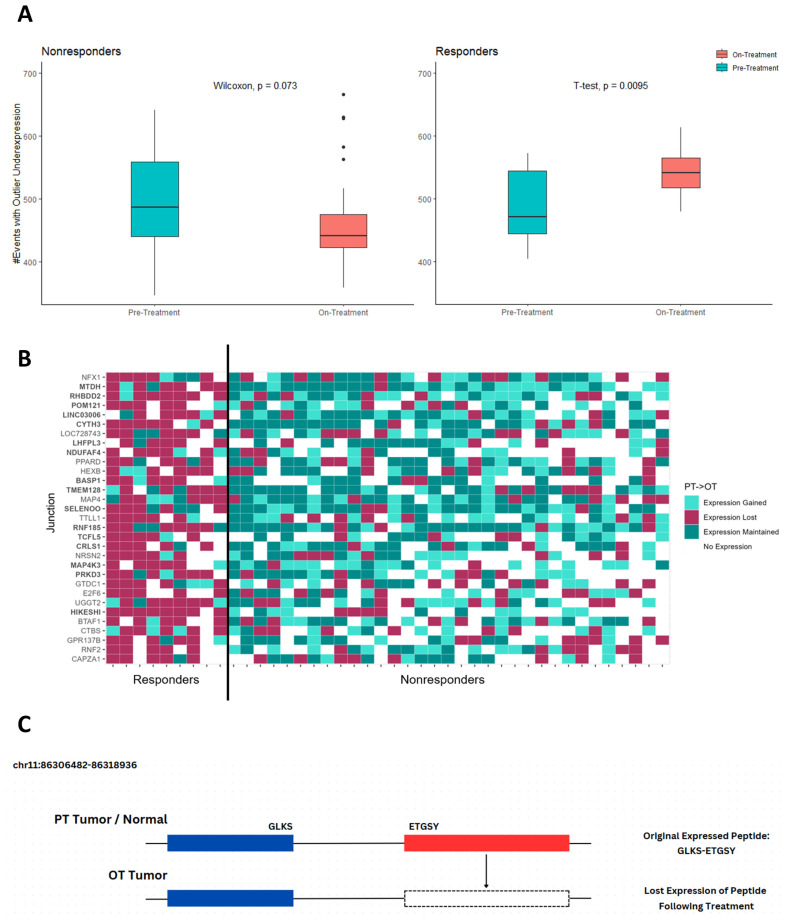
Lost expression following treatment. (**A**) Number of lost expression events detected by OutSplice analysis on pre-treatment (PT) and on-treatment (OT) melanoma biopsies for anti-PD1 responders (n = 9) and nonresponders (n = 33). The samples were biopsied 1–7 days before the first dose, and then on cycle 1, day 29 of treatment, as per Riaz et al. [26] (**A**) No significant change was seen in the nonresponder biopsies. Responder patients’ on-treatment biopsies were shown to have a significantly higher number of underexpressed insertion events compared to their pre-treatment counterparts. (**B**) Heatmap of the 31 events undergoing lost expression in the melanoma dataset. Colors represent how junction expression changes following treatment with anti-PD1 immunotherapy. Bolded events indicate those with a significant relationship between response and if expression was lost (*p* < 0.05) under the Fisher exact test. (**C**) Example of a lost expression event on the HIKESHI gene in a responder’s tumor appearing after anti-PD1 treatment. Black lines indicate introns and blue regions indicate the known exons. The red box represents an inserted sequence that is expressed outside of an annotated exon in both the normal and responder’s pre-treatment samples of which the expression is lost after anti-PD1 treatment.

Both OutSplice and SINE were run on a melanoma patient cohort of 9 responders and 33 nonresponders whose tumors were sampled before and after the first dose of nivolumab during anti-PD1 treatment [26]. OutSplice documented three types of splicing events: events with expression outside of known exons (Insertions), events without expression (Deletions), and events where a known exon is ignored in favor of a neighboring exon (Skipping). While no real change was seen in the responders regarding the number of deletions and skipping events after treatment, OutSplice found that responding patients showed a significant change in insertion events, which were underexpressed compared to normal tissue (Figure 1A). Further analysis demonstrated that these splicing events correlated with inserted sequences that were eliminated in responders as a result of treatment (Figure 1C). This underexpression after treatment, therefore, represented a loss of aberrant junction expression in responders. Of these events, we then narrowed down 31 of them of which the lost expression was shared across a majority of responders after exposure to immunotherapy. We found that nonresponders tended to maintain the expression of these events instead (Figure 1B). The Fisher exact testing then revealed that expression is lost significantly more often in the responders for these 31 events as a whole (*p* = 2.2 × 10^−10^), with 16 of the events being significant individually (*p*-adjusted < 0.05).

After SINE analysis, 65% of the 31 events that were lost in a majority of the responders showed a PHBR score of less than 2 for MHC-I, indicating them as binders. From these events we also identified 4 where MHC-I binding existed in the responders, but no binding ability was seen in the nonresponders. Additionally, one strong binder found in the responders only displayed weak binding in the nonresponders (Table 3). MHC-II binding affinity was also calculated for this dataset; however, only four events showed overall binding, with two having stronger affinity in the responders relative to the nonresponders (Table S2).

XCell analysis revealed that nonresponders tended to have lower CD4+ T-Cells (Median Infiltration = 2% vs. 13%, *p* = 0.02, *p*-adjusted = 0.25) and CD8+ central memory T cells (median infiltration = 2% vs. 13%, *p* = 0.04, *p*-adjusted = 0.32) at baseline (Table S3). Tumor purity analyses then further revealed that responders displayed significantly lower tumor purity scores after treatment compared to the nonresponders (Median Purity = 33% vs. 67%, *p* = 6.7 × 10^−3^, *p*-adjusted = 8.9 × 10^−3^). No significant difference in tumor purity was seen between the two groups before treatment (median responder vs. nonresponder purity = 56% vs. 75%, *p* = 0.11, *p*-adjusted = 0.14).

### 2.4. TCGA-SKCM Cohort

SINE was run using 361 junction events found across 5 TCGA-SKCM samples. Here, we found 11 peptides with immunogenic PHBR scores (<2) that were an exact match to the MS-validated peptides detailed in Li et al. (Table 4) [23]. Overall, based on SINE’s detection methods, 87 neojunctions were flagged as potentially immunogenic based on a PHBR score < 2 (Table S4).

## 3. Discussion

To demonstrate SINE’s ability to identify junction neoantigens in patient tumors, we applied our tool on 344 significant splicing alterations in tumor tissue compared to normal across 261 genes from a previously published dataset of 47 OPSCC tumors and 25 controls [25,28]. Altogether, we ended up detecting 125 potentially immunogenic events; 19 of which were very strong MHC-I binders. From this, we found two strong and six weak MHC-I binding peptides formed as a result of alternative splicing with a corresponding non-binding WT alternative, and a median occurrence value of 98%. Additionally, all of these splicing events displayed outlier overexpression relative to normal tissue in at least 30% of tumors, with seven of the events being overexpressed in at least 50%. Given the heterogeneity that is normally seen when comparing tumors, the identification of common highly expressed splicing event targets such as these could be beneficial for the development of generalized enhancing adjuncts to immunotherapy treatment [29].

However, not all of these events displayed an alternative WT junction-spanning peptide for comparison, which does not obviate their role as potential neoantigens. This lack of identifying a corresponding WT alternative can be attributed to a few potential reasons: the lack of an alternative junction event, the lack of alternative event expression, or the presence of a stop codon preventing junction-spanning peptides to be produced. While the first two reasons indicate that there is no available alternative being expressed, the splice event in question still undergoes differential expression in tumor tissue. Regarding the third reason, the inability of the WT to produce any meaningful peptide due to stop codons may indicate an overall frame shift occurring in the patient tumor that changes the culminating peptide. For these reasons, while not all of these events had a corresponding alternative WT junction, these events still warrant further evaluation as the expression of these events in the tumor relative to normal tissue may still be indicative of a biomarker for immunogenicity analysis [11].

Many of these genes flagged by SINE as potential neoantigen producers have not only been shown to have splice derived oncogenic isoforms in a variety of cancers, but also were notable as potential oncogenic or immunogenic targets. Mogilevsky et al. demonstrated how the overexpression of the *MKNK2b* isoform acts as a pro-oncogenic factor in glioblastomas, and were able to inhibit tumor growth through splicing manipulation towards the *MKNK2a* isoform instead [30]. Interestingly, *MKNK2b* was also found to be overexpressed in our tumor data, so its ability to act as a potential immunogenic target may provide a dual target for tumor control. Likewise, while developing a prognostic model to help predict the overall survival of cholangiocarcinoma patients, Lin et al. discovered a tumor up-regulated exon skipping event on the *SLC46A* gene, noting its relevance as a therapeutic target for anti-PD1 treatment [31].

While not splice-derived, other known oncogenes that have been noted as novel biomarkers that have been tagged by SINE include *FGFR1OP*, *INO80*, *PTPN18*, and *YKT6* [32,33,34,35]. Of these, *PTPN18* overexpression is particularly noteworthy as this gene has been shown to promote glioblastomas by decreasing CD8+ T cell infiltration to enhance immune suppression, making the identification of immunotherapy targets for this gene crucial for halting tumor growth [34]. Specifically, regarding head and neck cancer, elevated levels of the *YKT6* gene were associated with aggressive oral squamous cell carcinoma, matching what we see in our oropharyngeal cohort. Here, they noted how this gene’s upregulation gives rise to tumor immune evasion and the degradation of MHC-I, whereas decreased expression results in increased CD8+ T cells, making this gene a biomarker candidate [35]. The identification of a high-binding-affinity peptide on this gene using SINE suggests a potential target for immune-based therapies in aggressive cancers.

While SNAF was able to find more candidate targets in our OPSCC dataset, SINE may prove more useful and faster for those who have already narrowed down a list of potential alternative splicing targets. Additionally, SINE is capable of analyzing the potential neoantigen k-mers of a length of 8–11 amino acids, whereas SNAF is restricted to those of a of length 9–10. By considering the entire optimal binding length of MHC-I, the user will have more confidence in the actual absence of neoantigens along the splice region [36]. SNAF will also focus on reporting any potentially immunogenic peptides based on a three-frame in silico translation, whereas SINE will report the best potential immunogenic peptides based on the best alignment per sample, which may be useful to those seeking to take any potential sample-specific frame shift mutations into account. Further differences between the two software also include the definition of WT. SNAF’s method of filtering immunogenic events based on the number of junction spanning reads in the tumor compared to the normal is critical. However, it is also important to consider the potential alternative peptides that can be formed from highly expressed junction events in the normal tissue that share a start or end of the alternatively spliced event. In doing so, we can determine if the alternative spliced form in the tumor is actually novel and more likely capable of undergoing elimination by the immune system given the lack of an alternative binding possibility.

Regarding ASNEO, one potential explanation for the lack of overlapping results could be due to ASNEO’s requirement for the old hg19 build. This could also be due to differences in ASNEO’s classification methodology. ASNEO is capable of including other metrics for scoring neoantigen potential such as the peptide cleavage probability, and the T cell recognition score, which can act as a different, but effective means for evaluating MHC-I binding effectiveness [21]. However, unlike SINE, ASNEO is unable to work with mouse genomes, a feature that would be of benefit to many researchers given the cost/difficulty some may have obtaining human patient data.

Combined analysis with OutSplice and SINE were able to identify significant differences in splice junction expression in melanoma patients responding to anti-PD-1 immunotherapy compared to those that did not respond based on paired biopsies. Notably, these algorithms were able to identify a markedly decreased expression of immunogenic splicing events in response to treatment, a phenomenon that was highly specific and predictive for responders (Figure 1). This indicates that inside of the responding patient’s tumor, junction event expression is the same relative to a normal sample only before immunotherapy but is then lost upon receiving their first dose. Whereas in the nonresponders, the overall junction expression is either maintained or actually gained while on treatment.

To better understand these stark differences in junction expression between responders and nonresponders, we ran SINE on each of these events with lost expression in the responders and compared them to the nonresponders. We hypothesized that immunoediting could play a role in eliminating expression of these aberrant splice junctions, particularly any potentially immunogenic peptide along the splice junction or any additional novel insertion (Figure 1C). For each event, we then compared the average PHBR of responders losing expression during treatment against all nonresponders who maintained expression. In this study, we found 5 MHC-I binders with a stronger binding affinity in the responders compared to the nonresponders, as well as a significantly smaller tumor purity score following treatment. This indicates a trend toward responders having a higher immune infiltrate and provides some support that a boosted immune system will strengthen the body’s ability to eliminate the peptides produced from these regions.

This display of MHC-I/II binding peptides but lack of immune response in the nonresponding patients could also be due to the tumor microenvironment generating dysfunctional T cells in the nonresponders, reducing the effectiveness of the overall treatment. Another reason for nonresponse despite antigen presentation to MHC-I could be due to the differences in immune infiltration that existed prior to treatment. XCell analysis revealed smaller traces of both CD4+ T cells and CD8+ central memory T cells in the nonresponders’ tumors. These “cold tumors” will therefore be unable to trigger any immune response in these patients [37]. Due to the low numbers of responders, the power of this statistical analysis was likely limited, resulting in insignificant adjusted *p*-values. However, the uncorrected values do reveal this trend in immune infiltration.

It should also be noted that for SINE to detect some of the peptides in these regions, read duplication was required to allow for a successful assembly by Trinity, which may have resulted in an increase in the false positive rate of our experiment. Further, the number of paired pre-treatment to on-treatment tumor samples was limited to nine responding patients, so higher percentages may simply be due to a diminished sample size rather than a true effect. Future experiments will need to be carried out with melanoma responders and nonresponders at a greater sequencing depth in a sufficient number of samples along these regions. However, despite the lack of a clear difference in PHBR scores for all of our selected events, researchers should find SINE’s capability to use both generalized and splice-derived regions of interest beneficial for calculating the differences between responders and nonresponders.

To help evaluate SINE’s efficacy, we further tested our pipeline against a subset of the TCGA-SKCM ground-truth dataset. After running SINE against 361 neojunctions of which the existence was validated with MS evidence, we found that SINE was able to successfully recognize 87 of these events as immunogenic. Additionally, from these events, SINE picked up the exact neoantigen/peptide match in 11 events. These data demonstrate our tool’s ability at identifying the existence of true clinically relevant neojunctions. The major reasoning for why all neojunctions were not picked up by SINE as immunogenic and why the peptides were not found in all samples is most attributed to the frame selection step where only the best aligned reading frame per sample is utilized. This is carried out in an attempt to get the most accurate truly formed isoform per sample as opposed to considering all frames as only one frame will actually produce peptide. So, while a particular patient may display reads mapping across a splice event, it may not actually produce a relevant neoantigen that another patient displays due to the possibility of a frame shift. This makes the identification of the true isoform on a per patient level a critical component in the detection of neoantigens.

Through the use of SINE, we were able to document several top scoring strong and weak MHC-I/II-binding peptides that span aberrant splice junctions in head and neck cancer patient tumors. Additionally, we show how SINE can be used to document the differences in immunogenicity between anti-PD1 responders and nonresponders in a melanoma dataset. In accordance with these results, SINE was able to demonstrate the potential for these peptides to act as neoantigen targets through the use of PHBR analyses and the evaluation of the corresponding non-MHC-I/II binding WT junction spanning peptides. However, a future wet-lab validation using T-cell assays such as ELISpot will need to be carried out to confirm the existence of these peptides as true neoantigens. By being able to consider the binding affinity of all HLA alleles while also having compatibility with both mouse and human genomes, we believe researchers will find SINE a useful tool for the identification of potential neoantigens in their own independent datasets.

## 4. Materials and Methods

### 4.1. Data Preparation

The 47 primary OPSCC tumor samples and 25 oropharynx mucosal normal tissue samples were collected, prepared, and sequenced as previously detailed (OPSCC dataset) [28]. Paired-end sequencing and read trimming resulted in 80 million 100 × 100 base-pairs per sample. The resulting FASTQ data were then aligned using STAR (v.2.7.1a) with 2 Pass Mapping to the Human Genome Build 38 (hg38) [38]. Data were then also aligned to hg19 for downstream use with ASNEO. OutSplice was then run as previously described, resulting in 261 genes with 344 significant splicing events [25]. The 344 detected junction events for each of these genes were then used as the input into the SINE pipeline.

The 84 melanoma samples used in this study consisted of 9 anti-PD1 responding patients’ and 33 nonresponding patients’ pre-nivolumab and post-nivolumab treatment biopsies originally sequenced in a study performed by Riaz et al. (Melanoma dataset) [26]. 106 normal melanocytes were used to detect junction event removal in the tumor sample junctions with outlier underexpression and were collected and sequenced as previously described [39]. Adapter trimming was performed using BBMap [40]. Using OutSplice, splice events that existed prior to treatment, but were lost following initial treatment in a majority of responding patients were then provided to SINE’s pipeline with the 42 pre-treatment biopsies.

For evaluating SINE, the 5 primary solid tumor samples with the greatest number of neojunctions having MS evidence in the TCGA-SKCM dataset as evaluated by Li et al. were selected [23]. These samples’ 361 validated neojunctions were then provided to SINE. HLA-typing for all patients in all datasets was performed using HLA-HD (v.1.4.0) [41].

### 4.2. Computational Resources

SINE, ASNEO, and SNAF were run on the National Resource for Network Biology (NRNB) and the Triton Shared Computing Cluster (TSCC) hosted by the San Diego Supercomputer Center (SDSC) [42]. Parallel computing was used with the Simple Linux Utility for Resource Management (SLURM) system to process multiple samples and junction events at once. SINE was run with a maximum allocation of 4 CPUs and maximum memory requirement of <1 GB per sample. ASNEO was run with a maximum allocation of 4 CPUs and a maximum memory requirement of 8.93 GB per sample. SNAF was run with a maximum allocation of 4 CPUs and a maximum memory requirement of <1 GB.

### 4.3. SINE Pipeline

**Figure 2:**
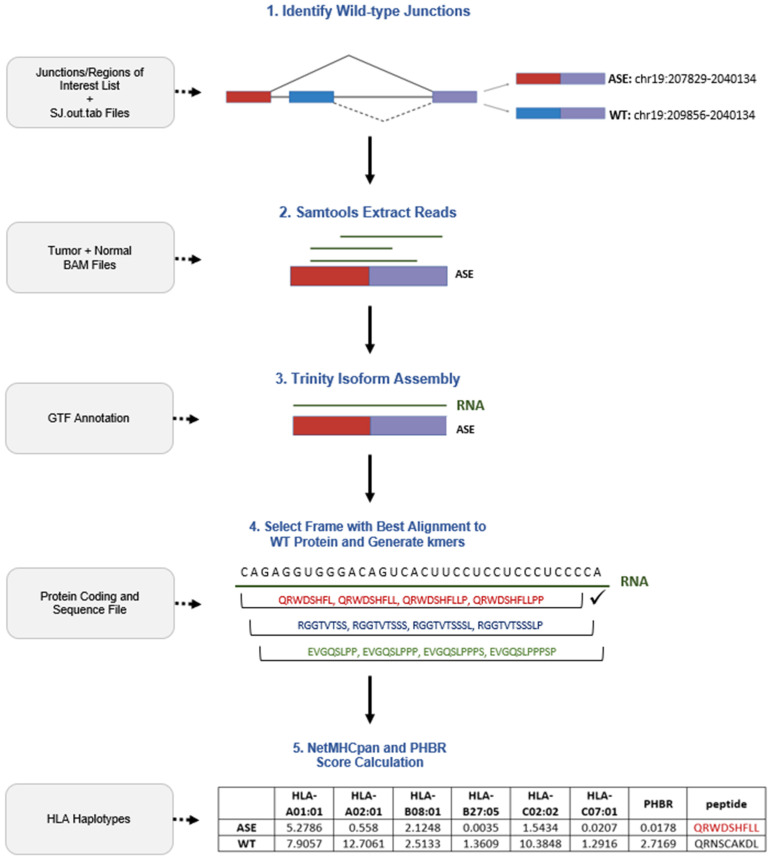
SINE workflow with an example splice event. Gray boxes indicate the required external input during each step. Right-facing arrows point toward a visualization of a SINE run on the detected event “chr19: 2037829–2040134”.

Figure 2 illustrates the workflow SINE follows for neoantigen detection. First, SINE uses STAR’s SJ.out.tab output and a directory of normal samples to identify the most commonly expressed junctions with a shared start or end coordinate/site for each provided alternative splicing event of interest (ASE). These junctions are then classified as wild-type (WT) junctions of which the binding score the ASE are compared to. Samtools (v.1.15.1) is then executed on the Binary Alignment Map (BAM) files for each tumor and normal sample to extract reads that span the junction of interest and their mates [43]. Trinity (v.2.11.0) then performs a de novo read assembly to identify all of the present isoforms in each sample [44]. After translating all the reading frames and retaining the contigs with the best alignment to the WT protein, k-mers of MHC I’s required binding size (8–11) that span the junction of interest coordinate positions are created. When selecting the contigs with the best alignment, the following parameters were used in the Biopython (v.1.81) local pairwise alignment module: 2 points for identical characters, −5 points for non-identical characters, −1 point when opening a gap, and −0.5 points when extending a gap.

For each splice event, every k-mer was run through NetMHCpan (v.4.1a) to calculate the binding affinity rank scores for every human leukocyte antigen allele (max: 6) in each patient [45]. Using a newly modified version of PyPresent, a PHBR score was calculated to predict the binding effectiveness of the junction spanning k-mers/peptides and records the potential neopeptide based on the strongest possible combination for each patient [24]. Events with a PHBR threshold < 0.5 and <2 were, respectively, designated as strong and weak MHC-I binders. Events > 2 were considered to be non-binders. To consider HLA type diversity across all patients expressing a particular ASE, an average PHBR score was also calculated for each detected event. WT PHBR scores were then calculated as the average score across each of the tumor sample’s HLA types. The most principal events with potential neoantigens were then selected from the results based on the following criteria: relevant binding score (<2 PHBR), identification of an alternative WT junction, those with outlier overexpression, and presence in a majority of tumor samples. This methodology was then repeated for MHC-II, with exceptions for a different binding size (15) and thresholds for relevant binding affinity (Strong Binders: <1 PHBR, Relevant Binders < 5 PHBR).

Alternatively, SINE can also use a region-of-interest workflow to detect immunogenic regions that not only span the junction of interest, but also those that lie upstream or downstream through the provision of generic coordinates. This feature was added to interrogate novel peptide sequences resulting from splicing events, including insertions or intron retention that would provide additional peptide diversity, instead of an exon-exon junction. When running SINE with this methodology, the same workflow was followed, except the WT junction identification step is skipped and instead of junction-spanning reads, reads that span any part of the provided region and their mates were extracted and assembled. SINE is open-source and is available on our GitHub (https://github.com/GuoLabUCSD/SINE, accessed on 21 October 2024).

### 4.4. Immunotherapy-Treated Melanoma Dataset

To understand the change in splice-derived neoantigens in response to immunotherapy, we ran OutSplice and SINE’s region of interest workflow to determine the presence of immunogenic regions. Splice junction expression was therefore evaluated before and after immunotherapy treatment in both responders and nonresponders. Following the OutSplice run on the Melanoma dataset, junction events in a majority of the responders that displayed outlier underexpression relative to normal tissue only after initial treatment (i.e., lost expression) were selected for use with SINE. Based on these junctions, the actual chromosomal regions were then manually selected based on the consensus region in responding patients’ pre-treatment samples where expression was lost following treatment. To help aid the Trinity assembly of isoforms, the number of reads spanning the region was duplicated 10-fold using SINE’s read boost function due to low read counts in this dataset. To compare responders and nonresponders, Pre-treatment samples with sequences that lead to a pre-mature stop codon on the event and a peptide shorter than the preferred MHC-I/II binding length (8–11, or 15), or samples where Trinity was unable to assemble an isoform, were excluded from the averages. PHBR averages were calculated using a subset of the responders where expression on the splice region was specifically lost and a subset of the nonresponders where expression was specifically maintained following initial treatment. Tidyestimate (v.1.1.1) was used for all tumor purity calculations [46,47]. XCell (v.1.0) was run to determine the cell types responsible for any differences in immune infiltration between responder and nonresponder tumors [48].

### 4.5. ASNEO Pipeline

Hg19-aligned data files were provided along with the corresponding reference genome FASTA file to the ASNEO python script (v.n/a) for analysis. Default settings were used for all samples, with the exception that the patient-specific HLA allele types were provided.

### 4.6. SNAF Pipeline

Similar to SINE, the BAM files used in the SNAF (v.0.7.0) pipeline were obtained through STAR alignment. Alternative splicing event quantification was performed using AltAnalyze with default settings as per the usage guide [23,49]. Our 25-oropharynx mucosal normal tissue samples were included as an additional control database to pair along with the SNAF default GTEx database of ~2500 normal samples spanning 54 different tissue types. Default settings were then used for all samples, with exception for the patient specific allele types and the number of CPU cores.

### 4.7. Statistical Analysis

Statistical analyses were conducted using SciPy (v.1.11.3), Python (v.3.10.13), and R (v.4.3.1) [50]. Shapiro–Wilk normality tests were performed on the responders and nonresponders pre-treatment and on-treatment groups. Normality testing revealed that all groups, except for the nonresponder’s on-treatment group, followed a normal distribution. Therefore, the statistical differences between the groups were then assessed using the nonparametric Wilcoxon signed-rank test for the nonresponders, but a parametric paired samples t-test for the responders. Statistical differences between responders and nonresponders were assessed using Fisher’s exact test to compare the number of samples with expression loss on the detected splice events. Tumor infiltration analyses comparing responders to nonresponders, and pre-treatment to on-treatment were carried out using Wilcoxon rank sum tests and Wilcoxon signed-rank tests respectively. *p*-value adjustments for the tumor infiltration tests and for the Fisher exact analyses were performed using the Benjamini–Hochberg method. All tests were performed using an alpha level of 0.05.

## Figures and Tables

**Table 1:** Summarized SINE results for the OPSCC dataset. PHBR columns represent the average for all samples containing a potential peptide at that event (ASE) and its corresponding wild type (WT). Best peptide/HLA combination represents the junction-spanning peptide with the strongest binding and which HLA type it binds to. Relevant binding is defined as splicing events in the gene where the average PHBR score was <2. %Samples indicates the percentage of tumors that both express an event and display a junction-spanning peptide of appropriate MHC-I binding length. %Tumors w/Outlier Overexpression (OE) represents the percent of tumors that specifically displayed outlier overexpression of the event. Events in bold represent the ASEs with MHC-I binding and a WT event that shows no binding efficacy.

	ASE PHBR	WT PHBR	Best Peptide/HLA Combination	%Samples (x/47 × 100)	Gene Symbol	%Tumors w/Outlier OE (x/47 × 100)
**chr19: 2037829–2040134**	**0.38**	**3.12**	**QRWDSHFLL B27:05**	**100%**	***MKNK2b***	**61.7%**
chr9: 136792030–136792251	0.43	0.44	VVAGSVVSY A26:08	100%	*TMEM141*	57.4%
**chr6: 167010893–167013508**	**0.45**	**4.21**	**TTPSKIPRY A26:08**	**97.9%**	***FGFR1OP***	**78.7%**
**chr6: 167003811–167004264**	**0.55**	**3.87**	**VFQPETSTL C14:02**	**100%**	***FGFR1OP***	**83%**
chr9: 4727924–4740937	0.61	1.34	ASLFPETQQY B15:01	66%	*AK3*	40.4%
**chr16: 29996617–29996808**	**0.7**	**4.09**	**SFLLDRLLQY A29:02**	**100%**	***INO80E***	**44.7%**
chr14: 105491522–105491622	0.74	1.12	RPEATSAL B07:02	91.5%	*C14orf80*	59.6%
chr3: 158649157–158650005	0.79	1.34	YFDGDFGHF C04:01	61.7%	*GFM1*	31.9%
**chr17: 28399713–28400610**	**1.15**	**4.13**	**AVLIGMLEK A11:01**	**91.5%**	***SLC46A1***	**48.9%**
**chr2: 130359492–130359608**	**1.21**	**2.44**	**WEFGVKVIL B40:01**	**89.4%**	***PTPN18***	**51.1%**
**chr9: 34665680–34665978**	**1.33**	**3.09**	**SQPPLQETF B15:01**	**59.6%**	***LOC730098***	**55.3%**
**chr7: 44208198–44211023**	**1.35**	**3.54**	**LHNTMESLL B38:01**	**97.9%**	***YKT6***	**48.9%**

**Table 2:** Shared splicing event detections between SINE and SNAF.

	Reported SINE Peptide(s)	Reported SNAF Peptide(s)	Gene Symbol	SINE ASE PHBR Score
chr7: 100199367–100199541	LLLEKDQNL	LLLEKDQNL, SLLLEKDQNL, NLGDVQESTL	*STAG3*	1.41
chr7: 22485060–22492565	REFHYIQRL, FHYIQRLL	REFHYIQRL, EFHYIQRLL, WREFHYIQRL	*STEAP1B*	0.72
chr17: 62680062–62680170	GPRGVTRPPF, VTRPPFSY, TRPPFSYHNF	PAPVLLPQF, SERGHPAPV, SERGHPAPVL, ERGHPAPVL, RGHPAPVLL	*MRC2*	0.39

**Table 3:** SINE results per gene for the regions of interest in the melanoma dataset. The responders’ column indicates the average PHBR score across anti-PD1 responders that lost the expression of the splice event after initial treatment. Responders’ Best peptide/HLA combination represents the junction-spanning peptide with the strongest binding and which HLA type it binds to. The nonresponders column indicates the average PHBR score across anti-PD1 nonresponders that maintained the expression of the splice event after initial treatment. Events labeled * contained a pre-mature stop codon, preventing the formation of a relevant peptide for PHBR analysis. Events in bold indicate those with strong/weak MHC-I binding affinity in the responders, but only weak/non-binding affinity in the nonresponders.

	Responders’ PHBR	Responders’ Best Peptide/HLA Combination	Nonresponders’ PHBR	Gene Symbol
chr1: 84563245–84563256	0.04	YYNYKVRLF HLA-C07:51	0.04	*CTBS*
chr4: 4237071–4237077	0.05	HPILRSAAL HLA-B35:03	0.06	*TMEM128*
chr7: 75881487–75881490	0.3	LPSEVVYRL HLA-B35:01	0.4	*RHBDD2*
**chr5: 74700753–74700851**	**0.44**	**SLQPPPLRFK HLA-A03:01**	**1.57**	***HEXB***
chr20: 6006807–6006834	0.47	IKYENPWTI HLA-C06:02	0.49	*CRLS1*
chr2: 37317807–37317899	0.56	RTWIGEIPY HLA-A32:01	1.99	*PRKD3*
**chr2: 11450008–11450011**	**0.58**	**VPAPREVGL HLA-B07:02**	**2.18**	***E2F6***
chr1: 112669896–112669918	1	NEYQGTQAY HLA-B44:03	0.77	*CAPZA1*
**chr22: 50204986–50205024**	**1.03**	**RQILGDPTY HLA-B15:01**	**9.15**	***SELENOO***
chr7: 72901070–72901128	1.09	NPPTTVSQI HLA-B51:01	0.69	*POM121*
chr1: 236207133–236207172	1.17	STEEKLGEY HLA-A01:01	0.96	*GPR137B*
chr22: 31176262–31176358	1.17	SPVSDSQLL HLA-B35:03	0.75	*RNF185*
chr9: 33338507–33338509	1.24	SLKSEADATF HLA-B15:01	1.72	*NFX1*
**chr2: 144229849–144229871**	**1.3**	**LFYRGSLYL HLA-A23:01**	**9.09**	***GTDC1***
chr11: 86318936–86318960	1.35	GLKSETGSY HLA-B15:01	0.89	*HIKESHI*
chr22: 43074660–43074673	1.67	QSLPMLPRL HLA-B57:01	NA	TTLL1 *
chr2: 39254521–39254545	1.68	IESIVIELF HLA-B44:02	0.79	*MAP4K3*
chr10: 91997080–91997108	1.73	KRWMKIKSV HLA-C06:02	1.14	*BTAF1*
chr7: 6257736–6257769	1.82	AAVPEDLSL HLA-C03:04	1.19	*CYTH3*
**chr8: 97696204–97696221**	**1.85**	**SIFSGIAAW HLA-A32:01**	**3.12**	***MTDH***
chr5: 17143578–17143591	2.27	EEANTCSFW HLA-B44:02	2.53	*BASP1*
chr13:95816081–95816104	2.34	KIQALQEAW HLA-A32:01	2.6	*UGGT2*
chr6: 35352929–35353001	2.58	NTKRRDSL HLA-B08:01	6.43	*PPARD*
chr20: 62843780–62843793	3.2	RFRETESGLEF HLA-A24:02	5.25	*TCFL5*
chr3: 47940517–47940534	3.39	GSDTTGESKSL HLA-C08:02	3.41	*MAP4*
chr6: 96897659–96897665	3.7	REQISRECL HLA-B40:01	2.24	*NDUFAF4*
chr7: 104740892–104740916	4.95	SRDQLGNMV HLA-B39:01	1.72	*LHFPL3*
chr1: 185091565–185091578	5.31	QEAITDGLEI HLA-B40:01	9.98	*RNF2*
chr7: 150408642–150408664	23.61	ELKETWRGHF HLA-B08:01	35.71	*LOC728743*
chr20: 348046–348063	30.87	RGSGELEGR HLA-A31:01	24.59	*NRSN2*

**Table 4:** SINE-detected neoantigens in the TCGA-SKCM dataset. The Exact Peptide Match column indicates the SINE-detected peptide previously documented as a neoantigen with MS evidence. Sample IDs indicate the case ID number from the Genomic Data Commons that SINE was able to find the peptide in. All peptides presented here were detected with an average PHBR < 2 across the samples listed in Sample ID.

	Exact Peptide Match	Sample ID	Gene Symbol
chr5: 90488129–90490593	ERQETGVLL	TCGA_BF_AAP1	*POLR3G*
chr1: 161710492–161710760	HAAASFETL	TCGA_BF_AAP1 TCGA_D9_A4Z2	*FCRLA*
chr12: 55956189–55956949	STESITATL	TCGA_BF_AAP1	*PMEL*
chr1: 179131481–179142933	ALPDLTEAL	TCGA_FR_A2OS	*ABL2*
chr20: 31797515–31798386	VAVQEPFQL	TCGA_BF_AAP1 TCGA_BF_A3DM TCGA_FR_A2OS TCGA_EB_A5VU	*TPX2*
chr18: 66509195–66511568	IIDNQEPVF	TCGA_D9_A4Z2	*CDH19*
chr15: 32811111–32857015	FQKGHPFPM	TCGA_BF_AAP1 TCGA_BF_A3DM TCGA_FR_A2OS TCGA_D9_A4Z2	*FMN1*
chr5: 33954504–33963931	FQTRRAMTL	TCGA_BF_AAP1 TCGA_EB_A5VU	*SLC45A2*
chr17: 79950575–79950756	RETDFKMKV	TCGA_FR_A2OS	*TBC1D16*
chrX: 54750106–54753651	IEDPKAGQF	TCGA_D9_A4Z2 TCGA_EB_A5VU	*ITIH6*
chrX: 9743655–9746044	VTAGRQGIY	TCGA_FR_A2OS TCGA_D9_A4Z2 TCGA_EB_A5VU	*GPR143*

## Data Availability

The OPSCC dataset presented in this study is available on request from the corresponding author. The data are not publicly available due to privacy restrictions, but are in the process of being added to a public repository. Previous RNA-Seq junction data are available in Gene Expression Omnibus (GEO) at GSE112026. The melanoma dataset sequenced by Riaz et al. is publicly available on GEO at GSE91061 [26]. The normal melanocytes were obtained through The Database of Genotypes and Phenotypes (dbGaP) and are available upon request at phs001500.v2.p1.

## Supplementary Materials

The following supporting information can be downloaded at: https://www.mdpi.com/article/10.3390/ijms26010205/s1.
