# Supplementary material for: Identifying Strong Neoantigen MHC-I/II Binding Candidates for Targeted Immunotherapy with SINE
Source: Int J Mol Sci. 2024 Dec 29;26(1):205. doi: 10.3390/ijms26010205 (PMC11720059; doi:10.3390/ijms26010205)
Supplement: Supplementary file 1 [file ijms-26-00205-s001.zip › Table S1.pdf]

Table S1: SINE results on the OPSCC dataset for all strong/weak MHC-I/II binders. PHBR columns represent the average for all samples containing a potential peptide at that event (ASE) and its corresponding wild-type (WT). Best peptide / HLA combination represents the junction spanning peptide with the strongest binding and which HLA-type it binds to. %Samples indicates the percentage of tumors that both express an event and display a junction spanning peptide of appropriate MHC-I/II binding length. Outlier Expression represents the percent of tumors that specifically displayed outlier overexpression (OE) or underexpression (UE) of the event relative to normal tissue.

	ASE PHBR	WT PHBR	Best peptide / HLA Combination	%Samples (x/47*100)	Outlier Expression	Gene Symbol
MHC-I						
Strong Binders						
chr1:161173727-161173859	0.13	NA	YSLPYPQYF C02:02	100.0%	OE	*B4GALT3*
chr6:35341020-35347067	0.19	0.58	KLLRVLYSV A02:01	2.1%	OE	*PPARD*
chr4:99318184-99318785	0.29	NA	KAYEVRIKM C12:03	44.7%	UE	*ADH1B*
chr13:98446817-98448236	0.32	NA	AESEYTFERW B44:02	95.7%	OE	*FARP1*
chr12:56475682-56475945	0.32	NA	AEKFDFVLQY B44:03	95.7%	OE	*GLS2*
chr2:99171429-99171510	0.34	1.9	PYIRHTHQL A24:02	89.4%	UE	*MITD1*
chr1:159073519-159113713	0.35	NA	RLLWKEIPVRL A02:01	2.1%	OE	*AIM2*
chr22:45418005-45418036	0.36	NA	QTNPTVVFF A32:01	12.8%	OE	*RIBC2*
chr19:48399145-48404734	0.37	NA	RTWAHSLQEK A03:01	36.2%	OE	*GRIN2D*
chr19:2037829-2040134	0.38	3.12	QRWDSHFLL B27:05	100.0%	OE	*MKNK2*
chr22:45402571-45403495	0.38	0.97	QPGQNVTRVSL B07:02	2.1%	OE	*SMC1B*
chr17:62680062-62680170	0.39	0.65	GPRGVTRPPF B07:02	27.7%	OE	*MRC2*
chr16:67656881-67657239	0.39	1.57	APAERPLRL B07:02	40.4%	UE	*CARMIL2*
chr11:65379561-65379867	0.4	0.29	YRHESLLGF C07:02	48.9%	UE	*SLC25A45*
chr9:136792030-136792251	0.43	0.44	VVAGSVVSY A26:08	100.0%	OE	*RP11-216L13.17*
chr2:241493444-241494008	0.45	NA	YTFERWMEV C15:02	80.9%	UE	*FARP2*
chr6:167010893-167013508	0.45	4.21	TTPSKIPRY A26:08	97.9%	OE	*FGFR1OP*
chr8:39914009-39914194	0.46	0.35	FALPNPQKW C12:02	27.7%	OE	*IDO1*
chr12:56471836-56472119	0.46	NA	AAAEGHIEV C02:06	89.4%	OE	*GLS2*
Weak Binders						
chr1:50421544-50423112	0.53	0.47	GPVMELRSEL B07:02	19.1%	OE	*DMRTA2*
chr1:31798323-31798442	0.55	NA	RDPRNLDLFL B07:02	34.0%	OE	*SPOCD1*
chr6:167003811-167004264	0.55	3.87	VFQPETSTL C14:02	100.0%	OE	*FGFR1OP*
chr10:4842504-	0.55	0.39	SHIKENIQA	25.5%	OE	*AKR1E2*

4908351			B39:06			
chr20:59922418-59932062	0.56	NA	SPEPAYLSV B51:01	6.4%	OE	*SYCP2*
chr7:40793501-40860316	0.56	0.45	YFIGPAVRY A29:02	17.0%	OE	*SUGCT*
chr5:171028989-171029442	0.58	0.72	SQSKVQMAW B44:02	2.1%	OE	*RANBP17*
chr7:107924389-107926183	0.6	NA	SAEDVKKTL C03:03	97.9%	OE	*LAMB1*
chr9:4727924-4740937	0.61	1.34	ASLFPETQQY B15:01	66.0%	OE	*AK3*
chr15:74902063-74902604	0.62	NA	RHYPPRLHL B14:01	4.3%	UE	*FAM219B*
chr11:117206798-117207067	0.63	2.22	RLLESAMSGK A03:01	100.0%	UE	*PCSK7*
chr19:4447625-4448318	0.64	NA	KLGVDTIAKY B15:01	95.7%	UE	*UBXN6*
chr12:40028509-40033303	0.65	0.88	NSLNMKLRY A29:02	2.1%	OE	*SLC2A13*
chr22:18205600-18896455	0.67	NA	YVLNEKNFQRY A29:02	4.3%	OE	*TMEM191B*
chr19:56817579-56817746	0.68	1.15	SAYPSTSRGLK A11:01	36.2%	UE	*PEG3*
chr16:29996617-29996808	0.7	4.09	SFLLDRLLQY A29:02	100.0%	OE	*INO80E*
chr7:22485060-22492565	0.72	0.89	REFHYIQRL B40:18	36.2%	OE	*STEAP1B*
chr3:189309928-189310386	0.73	1.11	SASPSKIMR A33:03	14.9%	OE	*TPRG1*
chr19:4446413-4446500	0.73	1.21	LRSEAVERL C06:02	97.9%	UE	*UBXN6*
chr14:105491522-105491622	0.74	1.12	RPEATSAL B07:02	91.5%	OE	*C14orf80*
chr2:130182452-130182627	0.78	3.46	AIDPDVFKI C05:01	100.0%	UE	*MZT2B*
chr3:158649157-158650005	0.79	1.34	YFDGDFGHF C04:01	61.7%	OE	*GFM1*
chr1:31793428-31793747	0.81	NA	RVPPSGLHV C01:02	27.7%	OE	*SPOCD1*
chr7:103312643-103312947	0.84	NA	KRYKELVEM B27:05	100.0%	OE	*DNAJC2*
chr6:85543306-85543605	0.86	NA	FIVEEIQRI A02:05	97.9%	UE	*SNX14*
chr9:136791424-136791711	0.86	0.81	HPGLGEYAA B56:01	100.0%	OE	*TMEM141*
chr4:22726478-22726541	0.86	NA	VHFKGAILF B38:01	95.7%	OE	*GBA3*
chr16:3065494-3065781	0.87	0.78	TSVSLGRPW B57:01	91.5%	OE	*IL32*
chr15:41842276-41842554	0.88	1.45	FPTSQEPLM B35:02	89.4%	UE	*JMJD7-PLA2G4B*
chr12:56472751-56473228	0.89	NA	IRNKTVVNL C06:02	89.4%	OE	*GLS2*
chr9:94094758-94095317	0.91	0.47	GPSEPVYFY B35:01	23.4%	OE	*PTPDC1*
chr5:148313399-148314100	0.91	8.98	KVDCSIYKK A11:01	21.3%	UE	*SPINK7*

chr2:74517004-74523100	0.91	NA	NQSEGKGSY B15:02	4.3%	OE	*TLX2*
chr1:161038075-161038551	0.93	0.8	IPVSELESA B56:01	100.0%	UE	*TSTD1*
chr2:58159800-58160108	0.93	NA	PYCSKPITL A24:02	93.6%	OE	*FANCL*
chr1:182939394-182939467	0.93	NA	ALIEERINL A02:01	51.1%	OE	*SHCBP1L*
chr12:56472195-56472690	0.96	NA	SALRRFAL B08:01	85.1%	OE	*GLS2*
chr7:100195382-100196300	0.96	0.88	HRYRDGVSL C07:01	25.5%	OE	*STAG3*
chr1:161674073-161675257	0.96	7.46	KRISALPGY B27:05	76.6%	UE	*FCGR2B*
chr19:7697075-7697236	0.99	1.18	KSQDLELSW B57:01	42.6%	UE	*FCER2*
chr20:59885509-59885928	1.01	1.7	KPVVQLSKL B07:02	42.6%	OE	*SYCP2*
chr7:100201866-100201949	1.02	NA	KSDASQKQL C05:01	83.0%	OE	*STAG3*
chr16:82164194-82170599	1.02	2.35	THVMKTKHF B15:18	44.7%	OE	*MPHOSPH6*
chr22:45383637-45386867	1.03	NA	RQKGKIFLL A32:01	12.8%	OE	*SMC1B*
chr5:150403312-150404680	1.04	0.63	KPTDAPPKVL B07:02	97.9%	OE	*CD74*
chr7:100100093-100100213	1.04	1.36	EKVKKFLQEF A25:01	10.6%	UE	*MCM7*
chr7:55950829-55953179	1.06	3.87	GDQSHVMSV B37:01	27.7%	OE	*RP11-15K19.2*
chr9:111756094-111756325	1.06	2.47	KLFERGWSA A02:01	12.8%	OE	*C9orf84*
chr15:91006729-91006950	1.07	1.66	RDVDFVTAL B37:01	93.6%	UE	*VPS33B*
chr2:169688948-169694261	1.09	NA	KESTVLGMSL B40:01	63.8%	OE	*CCDC173*
chr10:71740526-71740822	1.12	1.35	AQPRIQSEV B52:01	2.1%	OE	*CDH23*
chr16:4795858-4795948	1.14	NA	LTFMGTVLL B15:16	85.1%	OE	*SMIM22*
chr17:28399713-28400610	1.15	4.13	AVLIGMLEK A11:01	91.5%	OE	*SLC46A1*
chr3:197004180-197006549	1.17	1.44	VYGLLDKAQQF A24:02	27.7%	OE	*MELTF*
chr7:43647634-43648600	1.19	0.85	KYAGSRRSM C14:02	89.4%	OE	*COA1*
chr18:63960275-63961636	1.19	1.61	WVADKTKAW A25:01	40.4%	OE	*HMSD*
chr12:56474896-56475044	1.19	NA	YLKEKKCF B08:01	87.2%	OE	*GLS2*
chr2:130359492-130359608	1.21	2.44	WEFGVKVIL B40:01	89.4%	OE	*PTPN18*
chr10:96204088-96204532	1.21	5.32	FPPAQKQI B51:01	97.9%	UE	*BLNK*
chr12:56474720-56474846	1.26	NA	AALDLYFQL C03:03	80.9%	OE	*GLS2*
chr2:31405964-31496359	1.28	2.84	SRWWRKMQI C07:02	2.1%	OE	*XDH*

chr1:16976923-16978237	1.29	0.67	FLPDNPDYY A29:02	23.4%	OE	*MFAP2*
chr9:136791777-136791947	1.31	0.81	FVTGTGMAF C03:43	100.0%	OE	*TMEM141*
chr9:34665680-34665978	1.33	3.09	SQPPLQETF B15:01	59.6%	OE	*RP11-195F19.5*
chr19:4445623-4446049	1.34	NA	LVPSALLTF B46:01	100.0%	UE	*UBXN6*
chr7:44208198-44211023	1.35	3.54	LHNTMESLL B38:01	97.9%	OE	*YKT6*
chr1:54542874-54584655	1.36	1.27	EEVGLASVF B18:01	42.6%	OE	*ACOT11*
chr16:1456835-1457254	1.37	0.49	RDFKMESL B37:01	38.3%	OE	*CLCN7*
chr13:99969929-99970127	1.38	NA	KIHKRTHTV B08:01	23.4%	OE	*ZIC5*
chr19:4446920-4447550	1.38	3.76	VFQERINCL C14:02	97.9%	UE	*UBXN6*
chr17:75757310-75757416	1.39	NA	EPFLVDGPTL B35:02	100.0%	OE	*ITGB4*
chr14:103707215-103708522	1.4	NA	SSILTALQL C15:02	85.1%	OE	*XRCC3*
chr7:100199367-100199541	1.41	NA	LLLEKDQNL A02:01	87.2%	OE	*STAG3*
chr2:157556332-157566608	1.42	0.34	TRFSSITKC C06:02	2.1%	OE	*ACVR1C*
chr8:4994472-4994936	1.45	NA	LGPLSVAL C01:02	19.1%	OE	*CSMD1*
chr11:86950499-86951216	1.46	NA	NPLDTLCVI B51:01	10.6%	OE	*PRSS23*
chr1:153003296-153003321	1.47	0.35	KVPEPGYTK A11:01	38.3%	UE	*SPRR3*
chr6:1899796-1899896	1.48	NA	RRSPRGLRV C06:02	6.4%	OE	*GMDS*
chr19:18921964-18922326	1.49	NA	IKKIRFLVM B08:01	95.7%	UE	*DDX49*
chr2:68800044-68807273	1.5	3.14	TPKLRKKAV B08:01	31.9%	OE	*ARHGAP25*
chr16:89733412-89733482	1.51	NA	EREELPTIY B15:18	85.1%	UE	*ZNF276*
chr3:53865019-53868674	1.53	0.94	AEDQKQASW B44:03	14.9%	UE	*ACTR8*
chr7:2269639-2270012	1.53	1.74	SQDVQNKL C08:02	21.3%	OE	*SNX8*
chr12:56473320-56473463	1.56	NA	SFCQKLVSL C14:02	91.5%	OE	*GLS2*
chr12:56473594-56474544	1.57	NA	GQFAFHVGL B13:02	72.3%	OE	*GLS2*
chr13:98446205-98446666	1.58	NA	SHQDNHPLA B39:06	97.9%	OE	*FARP1*
chr16:3663562-3664274	1.62	0.49	AMKKKDTEV B08:01	97.9%	OE	*TRAP1*
chr9:35675567-35675761	1.63	NA	WRYGGDPPW B27:02	74.5%	OE	*CA9*
chr1:159196454-159196891	1.66	NA	GRGNPVPQQY B27:05	44.7%	UE	*CADM3*
chr2:235027855-235034871	1.67	NA	VRKKHIAEW C07:02	2.1%	OE	*SH3BP4*

chr16:69113542-69114324	1.69	2.22	GDVQHLYSL B37:01	21.3%	OE	*HAS3*
chr1:150511198-150511457	1.74	1.26	VWEEAMSRF C04:01	93.6%	OE	*ECM1*
chr12:75499951-75501723	1.75	NA	KPIVKPKEA B55:01	97.9%	OE	*KRR1*
chr1:161037826-161037913	1.76	NA	RSLGYTGAR A31:01	100.0%	UE	*TSTD1*
chr12:56471643-56471773	1.79	NA	KVNPFAKDR A31:01	87.2%	OE	*GLS2*
chr2:219182308-219182414	1.8	NA	SPETDSPQPL B07:02	74.5%	OE	*FAM134A*
chr7:151149142-151149648	1.81	NA	EPFSPRLSCQA B55:01	10.6%	OE	*GBX1*
chr18:23530297-23530387	1.81	1.56	DMLKRLSTA B08:01	100.0%	UE	*C18orf8*
chr7:99624180-99669095	1.81	5.17	SPEWLPQCL B35:03	46.8%	OE	*ZSCAN25*
chr4:15790918-15816511	1.83	1.63	SERHVDCQSVW B44:02	23.4%	OE	*CD38*
chr17:19745134-19745443	1.89	3.48	ALQASAMSK A03:01	38.3%	OE	*ALDH3A1*
chr20:17972751-17975684	1.89	0.97	DVFLQGKRF A25:01	76.6%	OE	*MGME1*
chr16:68986326-69022828	1.9	0.92	VVCHGDMVSK A03:01	4.3%	OE	*TANGO6*
chr3:127915909-127922950	1.9	NA	KEMFPDIFV B40:02	4.3%	OE	*KBTBD12*
chr15:91006445-91006652	1.91	1.66	GSVDFGPEV C02:06	95.7%	UE	*VPS33B*
chr17:40015514-40015691	1.96	NA	QSPMKLMAL C01:02	17.0%	OE	*CSF3*
MHC-II						
Strong Binders						
chr17:62680062-62680170	0.24	0.69	RPPFSYHNFDRSRHD DPA10201-DPB10402	27.7%	OE	*MRC2*
chr13:98446817-98448236	0.54	NA	ESEYTFERWMEVIRS DPA10202-DPB10401	95.7%	OE	*FARP1*
chr7:40793501-40860316	0.76	0.85	GPAVRYSKFKMSEAR DPA10201-DPB10201	17.0%	OE	*SUGCT*
chr6:85543306-85543605	0.85	NA	FDPFIVEEIQRIAEG DPA10202-DPB10401	97.9%	UE	*SNX14*
chr2:190250386-190252162	0.99	3.56	YRAGIATHFVDSEKR DQA10201-DQB10302	10.6%	OE	*HIBCH*
Weak Binders						
chr10:71740526-71740822	1.29	4.68	AQPRIQSEVVASDRG DPA10103-DPB13001	2.1%	OE	*CDH23*
chr11:61138835-61166490	1.31	6.33	EETLRPAAAQSEKEG DQA10103-DQB10302	6.4%	OE	*VPS37C*
chr12:75501816-75501923	1.5	NA	KRQKMEAIKAKQAEA DQA10103-DQB10402	97.9%	OE	*KRR1*
chr2:241493444-241494008	1.53	NA	ESKYTFERWMEVIQG DPA10202-DPB10401	80.9%	UE	*FARP2*
chr1:16976923-16978237	1.54	1.69	RAAYLFLLFLPDNPD DPA10201-DPB10401	23.4%	OE	*MFAP2*
chr22:45402571-45403495	1.55	6.17	DRAIAFQPGQNVTRV DRB11501	2.1%	OE	*SMC1B*

chr17:42573024-42573107	1.75	NA	FEEVGIETDEDYNVT DQA10501-DQB10201	80.9%	OE	*PSMC3IP*
chr12:56471836-56472119	1.89	NA	EGHIEVVKFLIEACK DPA10104-DPB11501	89.4%	OE	*GLS2*
chr1:16978314-16981514	1.94	5.51	SHKRNFSAVLSDTTP DPA10103-DPB10101	17.0%	OE	*MFAP2*
chr9:136792030-136792251	1.94	2.16	VAVVAGSVVSYGVTR DQA10505-DQB10601	100.0%	OE	*RP11-216L13.17*
chr11:117206798-117207067	1.95	21.89	YGSVWSAVDIRDRQR DQA10303-DQB10302	100.0%	UE	*PCSK7*
chr12:56475110-56475624	2.03	NA	GFSNATFQSEKETGD DQA10102-DQB10502	93.6%	OE	*GLS2*
chr7:103312643-103312947	2.12	NA	KELVEMVKAKKAAQE DQA10103-DQB10402	100.0%	OE	*DNAJC2*
chr19:4446413-4446500	2.24	5.38	TAEEIKREQRLRSEA DRB11103	97.9%	UE	*UBXN6*
chr10:26024087-26026372	2.28	1.78	QPELWSAEFNDFISN DPA10202-DPB10401	42.6%	OE	*MYO3A*
chr2:219182308-219182414	2.37	NA	GGPVETLSPETDSPQ DQA10201-DQB10302	74.5%	OE	*FAM134A*
chr16:28590666-28590773	2.37	NA	TNKYEVDDIDEEGKE DQA10301-DQB10201	95.7%	UE	*SGF29*
chr18:63960275-63961636	2.38	3.72	STTRVNSWVADKTKA DQA10201-DQB10402	40.4%	OE	*HMSD*
chr12:56471643-56471773	2.58	NA	AKDRWGNIPLDDAVQ DPA10103-DPB11101	87.2%	OE	*GLS2*
chr6:117538835-117539255	2.87	NA	KKKITGIRTTGSTQS DRB10402	100.0%	OE	*DCBLD1*
chr7:122011309-122012608	2.88	2.16	VNVVYSQTTQPVYNG DQA10201-DQB10503	25.5%	OE	*PTPRZ1*
chr22:45383637-45386867	2.93	NA	PERQKGKIFLLLQTV DPA10103-DPB10201	12.8%	OE	*SMC1B*
chr19:18921964-18922326	2.99	NA	IKKIRFLVMDEADRL DQA10505-DQB10501	95.7%	UE	*DDX49*
chr2:157556332-157566608	3.06	24.08	RNIRVGVTRFSSITK DPA10103-DPB10401	2.1%	OE	*ACVR1C*
chr6:75894840-75895231	3.07	11.9	DLALRSLDSYPVTSK DRB10102	61.7%	UE	*MYO6*
chr12:56473594-56474544	3.11	NA	SGQFAFHVGLPAKSA DRB10103	72.3%	OE	*GLS2*
chr19:4446719-4446836	3.11	4.49	QKVLLPAQDQEDPEE DQA10102-DQB10502	100.0%	UE	*UBXN6*
chr7:107929205-107929412	3.18	NA	EEAKRASKSATDVKV DPA10201-DPB11701	97.9%	OE	*LAMB1*
chr2:174335113-174336107	3.2	NA	GEEPRFGTTPLAMLA DPA10201-DPB11001	23.4%	OE	*SP9*
chr1:54542874-54584655	3.48	0.63	AGPARGFQSPQEEVG DQA10102-DQB10502	42.6%	OE	*ACOT11*
chr3:187241555-187243484	3.5	NA	EPYYKMLNNNTGIYT DRB10408	31.9%	UE	*MASP1*
chr20:17972751-17975684	3.6	0.79	DVFLQGKRFHEALES DPA10103-DPB10201	76.6%	OE	*MGME1*
chr16:3662967-3663424	3.66	3.92	VDHYKEEKFEDRSPA DPA10202-DPB10501	100.0%	OE	*TRAP1*
chr2:130182452-130182627	3.66	7.54	AGGAIDPDVFKILVD DPA10103-DPB10401	100.0%	UE	*MZT2B*
chr10:73234940-73235193	3.68	5.49	VEHVSTVGPQRQMKP DQA10303-DQB10603	95.7%	UE	*FAM149B1*

chr1:161037826-161037913	3.8	NA	SLGYTGARNYAGAYR DRB10407	100.0%	UE	*TSTD1*
chr7:100200969-100201090	3.92	NA	LEELLQSSFLDEDEV DPA10103-DPB10401	91.5%	OE	*STAG3*
chr7:151149142-151149648	3.93	NA	AEGKVYSSDEEKLEA DQA10201-DQB10202	10.6%	OE	*GBX1*
chr7:22485060-22492565	4.01	2.52	VSDSLTWREFHYIQR DPA10103-DPB10201	36.2%	OE	*STEAP1B*
chr15:90981981-90982554	4.1	1.86	FLFLVKTGFHHVGQE DPA10103-DPB10201	29.8%	OE	*PRC1*
chr1:159073519-159113713	4.23	NA	KEIPVRLIPKLSDGE DRB11454	2.1%	OE	*AIM2*
chr17:75757310-75757416	4.24	NA	TEPFLVDGPTLGAQH DRB10401	100.0%	OE	*ITGB4*
chr19:4447625-4448318	4.47	NA	VDTIAKYLDNIHLHP DQA10102-DQB10501	95.7%	UE	*UBXN6*
chr19:48399145-48404734	4.49	NA	DQSIGRTWAHSLQEK DRB10701	36.2%	OE	*GRIN2D*
chr12:56472195-56472690	4.65	NA	SALRRFALSAMDMEQ DPA10103-DPB11101	85.1%	OE	*GLS2*
chr16:69113542-69114324	4.7	10.71	DVQHLYSLADPQQVR DRB10404	21.3%	OE	*HAS3*
chr12:56475682-56475945	4.71	NA	FDFVLQYLNKMAGNE DRB11104	95.7%	OE	*GLS2*
chr6:126346318-126433551	4.88	5.63	INKEHVLAAAKVSEN DQA10505-DQB10601	51.1%	OE	*CENPW*
