# Supplementary material for: Identifying Strong Neoantigen MHC-I/II Binding Candidates for Targeted Immunotherapy with SINE
Source: Int J Mol Sci. 2024 Dec 29;26(1):205. doi: 10.3390/ijms26010205 (PMC11720059; doi:10.3390/ijms26010205)
Supplement: Supplementary file 1 [file ijms-26-00205-s001.zip › Table S2.pdf]

Table S2: SINE results on the Melanoma dataset for all strong/weak MHC-I/II binders. The responder's column indicates the average PHBR score across anti-PD1 responders that lost expression of the splice event after initial treatment. The nonresponder's column indicates the average PHBR score across anti-PD1 nonresponders that maintained expression of the splice event after initial treatment. Responders' Best peptide / HLA combination represents the junction spanning peptide with the strongest binding and which HLA-type it binds to. %Samples indicates the percentage of responder tumors that both express an event and display a junction spanning peptide of appropriate MHC-I/II binding length.

	Responders' ASE PHBR	Nonresponders' ASE PHBR	Responders' Best peptide / HLA Combination	%Samples (x/9*100)	Gene Symbol
MHC-I					
Strong Binders					
chr1:84563245-84563256	0.04	0.04	YYNYKVRLF C07:51	55.6%	*CTBS*
chr4:4237071-4237077	0.05	0.06	HPILRSAAL B35:03	11.1%	*TMEM128*
chr7:75881487-75881490	0.3	0.4	LPSEVVYRL B35:01	44.4%	*RHBDD2*
chr5:74700753-74700851	0.44	1.57	SLQPPPLRFK A03:01	55.6%	*HEXB*
chr20:6006807-6006834	0.47	0.49	IKYENPWTI C06:02	55.6%	*CRLS1*
Weak Binders					
chr2:37317807-37317899	0.56	1.99	RTWIGEIPY A32:01	11.1%	*PRKD3*
chr2:11450008-11450011	0.58	2.18	VPAPREVGL B07:02	55.6%	*E2F6*
chr1:112669896-112669918	1	0.77	NEYQGTQAY B44:03	55.6%	*CAPZA1*
chr22:50204986-50205024	1.03	9.15	RQILGDPTY B15:01	33.3%	*SELENOO*
chr7:72901070-72901128	1.09	0.69	NPPTTVSQI B51:01	44.4%	*POM121*
chr22:31176262-31176358	1.17	0.96	SPVSDSQLL B35:03	66.7%	*RNF185*
chr1:236207133-236207172	1.17	0.75	STEEKLGEY A01:01	55.6%	*GPR137B*
chr9:33338507-33338509	1.24	1.72	SLKSEADATF B15:01	44.4%	*NFX1*
chr2:144229849-144229871	1.3	9.09	LFYRGSLYL A23:01	11.1%	*GTDC1*
chr11:86318936-86318960	1.35	0.89	GLKSETGSY B15:01	88.9%	*HIKESHI*
chr22:43074660-43074673	1.67	NA	QSLPMLPRL B57:01	22.2%	*TTLL1**
chr2:39254521-39254545	1.68	0.79	IESIVIELF B44:02	55.6%	*MAP4K3*
chr10:91997080-91997108	1.73	1.14	KRWMKIKSV C06:02	55.6%	*BTAF1*
chr7:6257736-6257769	1.82	1.19	AAVPEDLSL C03:04	66.7%	*CYTH3*
chr8:97696204-97696221	1.85	3.12	SIFSGIAAW A32:01	66.7%	*MTDH*
MHC-II					
Weak Binders					

chr1:112669896-112669918	1.18	2.83	ENEYQGTQAYPLTIA DPA10202-DPB10401	55.6%	*CAPZA1*
chr4:4237071-4237077	3.04	1.08	EDHPILRSAALHSGG DQA10103-DQB10402	11.1%	*TMEM128*
chr7:6257736-6257769	3.59	8.59	SGVILAVAAVPEDLS DQA10301-DQB10302	55.6%	*CYTH3*
chr8:97696204-97696221	4.62	15.74	DRSIFSGIAAWSSVD DQA10505-DQB10301	66.7%	*MTDH*
