# Supplementary material for: Identifying Strong Neoantigen MHC-I/II Binding Candidates for Targeted Immunotherapy with SINE
Source: Int J Mol Sci. 2024 Dec 29;26(1):205. doi: 10.3390/ijms26010205 (PMC11720059; doi:10.3390/ijms26010205)
Supplement: Supplementary file 1 [file ijms-26-00205-s001.zip › Table S3.pdf]

Table S3: Immune Infiltration Analyses. xCell results represent differences between the responders and nonresponders pre-treatment biopsies. Tumor purity results represent the differences between the responders and nonresponders on-treatment biopsies.

	p-value	p-adjusted	Responder's Median	Nonresponder's Median
xCell Infiltration Analysis				
Adipocytes	0.55001301	0.71838433	0.1315	0.1071
Astrocytes	0.60229208	0.74023936	0	0
B-cells	0.07797329	0.34387368	0.17	0.1412
Basophils	0.34982558	0.5460692	0.0339	0
**CD4+ T-cells**	**0.01825971**	**0.25375167**	**0.1311**	**0.0237**
CD4+ Tcm	0.79443677	0.90792773	0	0
CD4+ Tem	0.27650926	0.47828628	0.1113	0.0053
**CD4+ memory T-cells**	**0.01481203**	**0.25375167**	**0.3907**	**0.2098**
CD4+ naive T-cells	0.34982558	0.5460692	0.0143	0.0013
CD8+ T-cells	0.2902647	0.48886686	0.1225	0.0565
**CD8+ Tcm**	**0.04306055**	**0.32224613**	**0.1332**	**0.0248**
CD8+ Tem	0.07052116	0.34387368	0.1178	0
CD8+ naive T-cells	0.10755038	0.34416123	0.0301	0.1189
CLP	0.06817093	0.34387368	0.1041	0.151
**CMP**	**0.01144197**	**0.25375167**	**0**	**0.0117**
Chondrocytes	0.84206963	0.93951608	0.0848	0.1225
Class-switched memory B-cells	0.23189905	0.46792009	0.114	0.0935
DC	0.10755038	0.34416123	0.2386	0.1834
Endothelial cells	0.98777156	1	0.1384	0.1595
Eosinophils	1	1	0.0873	0.0932
**Epithelial cells**	**0.01982435**	**0.25375167**	**0.0416**	**0.0677**
Erythrocytes	0.61301072	0.74023936	0	0
Fibroblasts	0.36584997	0.55669765	0.2126	0.2701
GMP	0.61301072	0.74023936	0.0608	0.0788
HSC	0.7475585	0.86988625	0.1203	0.2231
Hepatocytes	0.37403123	0.55669765	0.0374	0.0239
Keratinocytes	0.21443538	0.45746214	0	0
MEP	0.20333237	0.45746214	0	0.0425
MPP	0.20333237	0.45746214	0.2463	0.2142
MSC	0.11797695	0.35954881	0.1214	0.2179
Macrophages	0.25672134	0.46792009	0.2251	0.1966
Macrophages M1	0.08059539	0.34387368	0.0986	0.0692
Macrophages M2	0.91456147	0.97553224	0.1134	0.1263
Mast cells	0.72445304	0.85861101	0.0331	0.0328
Megakaryocytes	0.26320505	0.46792009	0.1804	0.1343
Melanocytes	0.33425387	0.5460692	0.3065	0.3381
Memory B-cells	0.05538605	0.32224613	0.0796	0.0456
**Mesangial cells**	**0.02425774**	**0.25874919**	**0.0905**	**0.0491**
Monocytes	0.43441393	0.61783314	0.1401	0.1355
Myocytes	0.95111493	0.99789107	0	0.0029
NK cells	0.20333237	0.45746214	0.1042	0.0759
NKT	0.19794049	0.45746214	0.2615	0.3882
Neurons	0.86611638	0.93951608	0.0325	0.0339
**Neutrophils**	**0.01144197**	**0.25375167**	**0.1326**	**0.0828**
Osteoblast	0.10755038	0.34416123	0.2983	0.3596
Pericytes	0.52974586	0.70632782	0.0269	0.0439
Plasma cells	0.61301072	0.74023936	0.0314	0.0279
Platelets	0.97554598	1	0.0373	0.0456
Preadipocytes	0.50007354	0.69428473	0.1178	0.1394
Sebocytes	0.50986535	0.69428473	0.0024	0.0039

Skeletal muscle	0.85407606	0.93951608	0.0082	0.0074
Smooth muscle	0.17254403	0.45746214	0.0544	0.1595
**Tgd cells**	**0.04631965**	**0.32224613**	**0.206**	**0.1286**
Th1 cells	0.26320505	0.46792009	0.0943	0.1937
Th2 cells	0.09786736	0.34416123	0.062	0.0254
Tregs	0.08889384	0.34416123	0.077	0.0115
aDC	0.39924658	0.5807223	0.2298	0.2221
cDC	0.19265396	0.45746214	0.1397	0.1122
iDC	0.12918046	0.37579769	0.1646	0.1255
ly Endothelial cells	0.26320505	0.46792009	0.1509	0.1116
mv Endothelial cells	0.21443538	0.45746214	0.2112	0.172
naive B-cells	0.23793874	0.46792009	0.0247	0.0156
**pDC**	**0.04466503**	**0.32224613**	**0.2181**	**0.143**
pro B-cells	0.05159606	0.32224613	0.0793	0.0126
Tumor Purity Analysis				
**stromal**	**0.00959176**	**0.00959176**	**869.089752**	**305.577983**
**immune**	**0.00667128**	**0.00889503**	**3300.58001**	**1121.55828**
**estimate**	**0.00667128**	**0.00889503**	**4265.39482**	**1584.11027**
**purity**	**0.00667128**	**0.00889503**	**0.33320297**	**0.6693103**
