# Supplementary material for: Identifying Strong Neoantigen MHC-I/II Binding Candidates for Targeted Immunotherapy with SINE
Source: Int J Mol Sci. 2024 Dec 29;26(1):205. doi: 10.3390/ijms26010205 (PMC11720059; doi:10.3390/ijms26010205)
Supplement: Supplementary file 1 [file ijms-26-00205-s001.zip › Table S4.pdf]

Table S4: SINE Results on the TCGA-SKCM Subsetted Dataset. SINE Best Binder indicates the best possible binding junction spanning peptide present at the potential neojunctions. Sample IDs indicate which samples SINE detected as expressing the event and displayed a junction spanning peptide of appropriate MHC-I binding length. Samples utilized included TCGA_BF_A3DM, TCGA_FR_A2OS, TCGA_BF_AAP1, TCGA_D9_A4Z2, TCGA_EB_A5VU. All splicing events listed here demonstrated a PHBR score < 2 in SINE.

	**SINE Best Binder**	**Sample IDs**	**Gene Symbol**
chr17:61245371-61368327	STFDRSVTL	TCGA_BF_A3DM<br>TCGA_FR_A2OS	*BCAS3*
chr12:52010562-52010881	LASQKVLTL	TCGA_BF_AAP1	*GRASP*
chr5:33947374-33951554	SVFSSLYSY	TCGA_BF_AAP1<br>TCGA_FR_A2OS<br>TCGA_D9_A4Z2<br>TCGA_EB_A5VU	*SLC45A2*
chr17:61354502-61368327	STFDRSVTL	TCGA_BF_A3DM<br>TCGA_D9_A4Z2	*BCAS3*
chr20:46726324-46726883	FGPARSTL	TCGA_EB_A5VU	*SLC2A10*
chr17:8207840-8210530	SALDPRLHL	TCGA_FR_A2OS<br>TCGA_EB_A5VU	*AURKB*
chr3:186105942-186161603	RRIPFPPEI	TCGA_FR_A2OS	*ETV5*
chr5:90488129-90490593	ERQETGVLL	TCGA_BF_AAP1	*POLR3G*
chr21:38122411-38156304	MAFVKHLL	TCGA_BF_A3DM	*DSCR8*
chr12:2861412-2864320	KVFGEQVVF	TCGA_BF_AAP1<br>TCGA_FR_A2OS	*FOXM1*
chr15:89215238-89217120	RAYELLRGY	TCGA_BF_AAP1<br>TCGA_BF_A3DM<br>TCGA_D9_A4Z2	*RLBP1*
chr20:49849680-49850762	IYFLGQGFF	TCGA_EB_A5VU	*SLC9A8*
chr6:70035939-70039282	ALPLSLTDL	TCGA_EB_A5VU	*COL19A1*
chr1:212421663-212444339	STNWPTLEF	TCGA_BF_A3DM<br>TCGA_D9_A4Z2	*NENF*
chrX:54753764-54753930	SVDGDPHFV	TCGA_FR_A2OS<br>TCGA_D9_A4Z2<br>TCGA_EB_A5VU	*ITIH6*
chr1:161710492-161710760	HAAASFETL	TCGA_BF_AAP1<br>TCGA_D9_A4Z2	*FCRLA*
chr12:55956189-55956949	STESITATL	TCGA_BF_AAP1	*PMEL*
chr1:179131481-179142933	ALPDLTEAL	TCGA_FR_A2OS	*ABL2*
chr1:214603321-214613714	FSRPREQVY	TCGA_EB_A5VU	*CENPF*
chr20:31797515-31798386	VAVQEPFQL	TCGA_BF_AAP1<br>TCGA_BF_A3DM<br>TCGA_FR_A2OS<br>TCGA_EB_A5VU	*TPX2*
chr14:102233789-102246057	IIPLQTIGL	TCGA_FR_A2OS<br>TCGA_D9_A4Z2<br>TCGA_EB_A5VU	*MOK*
chr19:40775211-40775611	VRGGPMPKL	TCGA_BF_A3DM	*MIA*

chr6:159803310-159803965	KYIAHSCNF	TCGA_BF_A3DM<br>TCGA_EB_A5VU	*PNLDC1*
chr2:112760764-112762503	NTKPYLKSK	TCGA_EB_A5VU	*CKAP2L*
chr6:10672636-10683187	NQDEDPLEV	TCGA_BF_AAP1	*C6orf52*
chr2:222201442-222201944	GQYGQSAFHY	TCGA_BF_AAP1<br>TCGA_BF_A3DM<br>TCGA_FR_A2OS<br>TCGA_D9_A4Z2	*PAX3*
chr21:44426736-44435131	GENPMGRTGL	TCGA_BF_A3DM<br>TCGA_FR_A2OS	*TRPM2*
chr15:32811111-32857015	FQKGHPFPM	TCGA_BF_AAP1<br>TCGA_BF_A3DM<br>TCGA_FR_A2OS<br>TCGA_D9_A4Z2	*FMN1*
chr1:26033315-26033723	ESFPERMVGF	TCGA_BF_AAP1<br>TCGA_BF_A3DM	*EXTL1*
chr1:67386669-67390029	YFQQKVFVL	TCGA_FR_A2OS<br>TCGA_EB_A5VU	*IL12RB2*
chrX:20241686-20266564	SAEDYIFIL	TCGA_FR_A2OS	*RPS6KA3*
chr5:33954504-33963931	FQTRRAMTL	TCGA_BF_AAP1<br>TCGA_EB_A5VU	*SLC45A2*
chr3:186105942-186108457	MTQEDPFSPR	TCGA_FR_A2OS	*ETV5*
chr12:51008624-51009117	STVDSLNSV	TCGA_BF_A3DM	*SLC11A2*
chr21:10542448-10543329	KESPHTSEF	TCGA_BF_AAP1	*TPTE*
chr10:126513939-126646565	GLRDSYHSR	TCGA_BF_AAP1<br>TCGA_D9_A4Z2	*C10orf90*
chrX:152728231-152732445	MSATTVSSL	TCGA_BF_AAP1<br>TCGA_D9_A4Z2	*CSAG1*
chr6:70035939-70039370	ALPELPGEASL	TCGA_EB_A5VU	*COL19A1*
chrX:152136969-152138475	EEAREVRTL	TCGA_BF_AAP1<br>TCGA_BF_A3DM	*MAGEA10*
chr6:33181170-33184145	ETAHSGAAA	TCGA_BF_AAP1<br>TCGA_FR_A2OS<br>TCGA_D9_A4Z2	*COL11A2*
chr11:33661014-33667873	SVPLPGYIEAY	TCGA_BF_AAP1<br>TCGA_BF_A3DM	*KIAA1549L*
chr5:134621001-134623962	LQFLGKTTL	TCGA_BF_A3DM	*SAR1B*
chr1:241867055-241872035	ELSEDDLLSQY	TCGA_BF_AAP1<br>TCGA_BF_A3DM	*EXO1*
chr2:96960440-96967520	EVPLTPEHR	TCGA_BF_AAP1<br>TCGA_BF_A3DM<br>TCGA_FR_A2OS	*FAM178B*
chr5:33964016-33982236	HYHALFTGF	TCGA_BF_AAP1<br>TCGA_FR_A2OS<br>TCGA_D9_A4Z2<br>TCGA_EB_A5VU	*SLC45A2*
chrX:154618273-154653247	RLLEFYLAM	TCGA_BF_A3DM	*CTAG1B*
chr10:126505085-126513848	KSDFTKETL	TCGA_BF_A3DM<br>TCGA_D9_A4Z2	*C10orf90*

		TCGA_EB_A5VU	
chr7:23317713-23319138	SRFAGASIKI	TCGA_D9_A4Z2	*IGF2BP3*
chrX:54774197-54788480	DIIGDVQIY	TCGA_FR_A2OS<br>TCGA_D9_A4Z2<br>TCGA_EB_A5VU	*ITIH6*
chr20:46358558-46358691	QPLRGVPAPA	TCGA_EB_A5VU	*SLC35C2*
chr6:395935-397111	SLPAQVHNY	TCGA_BF_AAP1<br>TCGA_FR_A2OS	*IRF4*
chr18:66509195-66511568	IIDNQEPVF	TCGA_FR_A2OS<br>TCGA_D9_A4Z2	*CDH19*
chr12:109557136-109561040	RLSDYLFTL	TCGA_BF_AAP1	*MMAB*
chr7:22494886-22500114	ARSGWLKPY	TCGA_BF_AAP1<br>TCGA_BF_A3DM<br>TCGA_EB_A5VU	*STEAP1B*
chr2:96967627-96970716	LLQEKREQAL	TCGA_BF_AAP1<br>TCGA_BF_A3DM<br>TCGA_FR_A2OS	*FAM178B*
chr2:96921262-96921478	REWPGKLQEL	TCGA_BF_AAP1<br>TCGA_BF_A3DM<br>TCGA_FR_A2OS	*FAM178B*
chr1:102979435-102984138	GRQGPKGSTGF	TCGA_FR_A2OS<br>TCGA_D9_A4Z2	*COL11A1*
chr17:44932242-44932673	EHLPSSPL	TCGA_BF_AAP1<br>TCGA_FR_A2OS<br>TCGA_D9_A4Z2	*KIF18B*
chr16:46689770-46690991	DMLRKAEEY	TCGA_BF_AAP1<br>TCGA_EB_A5VU	*ORC6*
chr19:3547521-3547848	TRLIVNLSQTY	TCGA_FR_A2OS<br>TCGA_EB_A5VU	*MFSD12*
chr14:67503551-67515086	TAPAPHRAV	TCGA_BF_A3DM	*TMEM229B*
chr6:26115135-26123528	AVTKYTSSK	TCGA_EB_A5VU	*HIST1H2BC*
chrX:54750106-54753651	IEDPKAGQF	TCGA_D9_A4Z2<br>TCGA_EB_A5VU	*ITIH6*
chrX:13934451-13938507	LQPWAQATL	TCGA_FR_A2OS<br>TCGA_D9_A4Z2<br>TCGA_EB_A5VU	*GPM6B*
chr2:224001922-224039081	YRLRGRPWW	TCGA_BF_A3DM<br>TCGA_FR_A2OS	*SERPINE2*
chr15:55270284-55270859	ARLWRKAKETK	TCGA_FR_A2OS<br>TCGA_D9_A4Z2	*RAB27A*
chrX:152716961-152717042	ALKEKICL	TCGA_BF_AAP1<br>TCGA_BF_A3DM	*MAGEA2B*
chrX:152727863-152728074	KRFPRQPKR	TCGA_BF_AAP1<br>TCGA_BF_A3DM<br>TCGA_D9_A4Z2	*CSAG1*
chr2:96960440-96970716	KRMLVEKY	TCGA_BF_AAP1<br>TCGA_FR_A2OS	*FAM178B*
chr20:63239233-63239412	ESAQEPGAR	TCGA_BF_AAP1<br>TCGA_BF_A3DM<br>TCGA_FR_A2OS<br>TCGA_D9_A4Z2<br>TCGA_EB_A5VU	*BIRC7*
chr5:16779645-	EFYLSTPENY	TCGA_FR_A2OS	*MYO10*

16780524		TCGA_EB_A5VU	
chr13:113044888-113045271	TSDARTGVI	TCGA_BF_AAP1<br>TCGA_BF_A3DM<br>TCGA_D9_A4Z2	*MCF2L*
chr10:100063725-100065128	KLAKVYSY	TCGA_BF_AAP1<br>TCGA_EB_A5VU	*CPN1*
chr3:48783086-48790544	NLDQLSQVL	TCGA_FR_A2OS	*PRKAR2A*
chr17:79950575-79950756	RETDFKMKV	TCGA_BF_AAP1<br>TCGA_FR_A2OS<br>TCGA_EB_A5VU	*TBC1D16*
chrX:141903978-141904717	SLQGAPESR	TCGA_D9_A4Z2	*MAGEC1*
chr1:165409648-165410702	VILLRAVSV	TCGA_BF_AAP1<br>TCGA_BF_A3DM<br>TCGA_EB_A5VU	*RXRG*
chrX:154652267-154652497	LLFIRLTAA	TCGA_BF_A3DM<br>TCGA_D9_A4Z2<br>TCGA_EB_A5VU	*CTAG2*
chr12:85286981-85301155	SYPQIQNNL	TCGA_BF_A3DM<br>TCGA_FR_A2OS<br>TCGA_D9_A4Z2	*ALX1*
chr15:48878712-48884248	ILTTNSRVL	TCGA_BF_A3DM<br>TCGA_FR_A2OS<br>TCGA_D9_A4Z2<br>TCGA_EB_A5VU	*SHC4*
chr22:22557580-22558971	ARSRDNFAVW	TCGA_BF_AAP1<br>TCGA_BF_A3DM<br>TCGA_FR_A2OS<br>TCGA_D9_A4Z2<br>TCGA_EB_A5VU	*PRAME*
chr22:46308207-46308319	SANEDDEVF	TCGA_BF_AAP1<br>TCGA_FR_A2OS<br>TCGA_EB_A5VU	*GTSE1*
chrX:149583388-149583547	SSEGSRDRL	TCGA_BF_AAP1	*MAGEA9B*
chrX:151671779-151672186	QEPCVAFNQQL	TCGA_BF_AAP1<br>TCGA_BF_A3DM	*PASD1*
chr13:111241336-111243872	GRKVVLQNI	TCGA_D9_A4Z2	*ARHGEF7*
chr18:74447283-74457062	ILAALCQDY	TCGA_BF_AAP1	*FAM69C*
chrX:9743655-9746044	VTAGRQGIY	TCGA_FR_A2OS<br>TCGA_D9_A4Z2<br>TCGA_EB_A5VU	*GPR143*
